# Detection method for transparent window cleaning device, image processing approach

**DOI:** 10.1038/s41598-022-07235-y

**Published:** 2022-02-25

**Authors:** Jiseok Lee, Hobyeong Chae, KyungMin Kim, Hwa Soo Kim, TaeWon Seo

**Affiliations:** 1 School of Mechanical Engineering, Hanyang University, Seoul, 04763 South Korea; 2 School of Mechanical System Engineering, Kyounggi University, Suwon, 16227 Korea

**Keywords:** Engineering, Mechanical engineering

## Abstract

Recent years, there has been an increase in the number of high-rise buildings, and subsequently, the interest in external wall cleaning methods has similarly increased. While a number of exterior wall cleaning robots are being developed, a method to detect contaminants on the exterior walls is still required. The exteriors of most high-rise buildings today take the form of a window curtain-wall made of translucent glass. Detecting dust on translucent glass is a significant challenge. Here, we have attempted to overcome this challenge using image processing, inspired by the fact that people typically use just the ‘naked eye’ to recognize dust on windows. In this paper, we propose a method that detects dust through simple image processing techniques and estimates its density. This method only uses processing techniques that are not significantly restricted by global brightness and background, making it easily applicable in outdoor conditions. Dust separation was performed using a median filter, and dust density was estimated through a mean shift analysis technique. This dust detection method can perform dust separation and density estimation using only an image of the dust on a translucent window with blurry background.

## Introduction

The number of high-rise buildings is increasing worldwide^1^. Accordingly, exterior wall cleaning robots are being increasingly studied^2–4^ all cleaning robots is their cleaning efficiency-that is, their ability to perform maximum cleaning with minimum action. Humans perform efficient cleaning by focusing more on very dusty windows and less so on windows with a smaller amount of dust. However, if a robot does not have a system with which to measure the amount of dust, it will always have to assume a worst case scenario when cleaning. When a cleaning robot has a system that can detect surface dust, it is able to adjust its cleaning method and various cleaning parameters based on the density of the dust, allowing it to achieve incremental cleaning efficiency.

Inspired by the fact that humans can see dust with their naked eyes, we attempted to solve the aforementioned problem through image processing. In this paper, we propose a simple method for estimating the degree of dust on glass using image processing on an image of dusty glass taken with a low-quality camera. There are several existing methods for detecting dust on a surface, but it is difficult to apply it to a window cleaning robot active on the exterior wall of a building. When detecting dust on a solar panel^5^, the background is very static, but when dust is detected on a translucent window, the background can be very dynamic. Dust detection methods in a robot vacuum cleaner sense the dust density in the air^6^, not on a surface, and thus such methods cannot be applied to dusty translucent glass. When measuring dust on a window using the reflections of IR rays^7^, there is a risk of inconsistent results-that is, depending on the sunlight conditions, there is a risk that incident IR is reflected off the glass surface, leading to different results.

The detection system itself should not be heavy as it must be installed on a mobile platform operating on the exterior wall of a building. It must be relatively resilient to external conditions such as brightness, and dust detection must be performed rigorously against a dynamic background reflected through the translucent glass. Dust density estimation of previous study^8^ was too sensitive to brightness condition, unable to be applied at real world. In this study, an image with a resolution of 720p is obtained using a camera, and the estimated translucent glass dust density is obtained using only image processing. A deep learning approach was excluded because the computing device is not sufficiently compact or energy efficient, which affects the robot’s operating time. This study is an extension of existing façade contaminant detection studies [8, 9] applied to the façade cleaning robot named ‘M1’^9–11^. The detection method was used on the exterior wall of an opaque building, making it difficult to deal with the situation of a translucent window. Therefore, a dust detection method in a windowed environment was studied. In addition, the robot manipulator has a cleaning module structure that constantly controls its distance to the wall through force control, making it suitable for use in this study.

The remainder of this paper is organized as follows. Section “Overall two-stage structure” describes the overall two-stage structure of our proposed method. The first is a dust separation stage that separates dust binary images from the original images. The second is a dust density estimation stage that estimates the true density of dust from separate dust binary images. Section “Test environment” describes the test bench and test environment used to determine the detection accuracy. Section “Dust separation stage” describes the first stage-that is, the Sobel^12^, Prewitt^13^, Median^14,15^ and Gaussian^16^ filter detection performance comparison results. The dust pixel separation procedure is also introduced. Section “True density estimation stage” describes the second stage-that is, the detection area proposal and dust density estimation process through mean shift clustering^7,17^. Finally, our discussion and conclusions are summarized in “Conclusion & discussion”.

**Figure 1:**
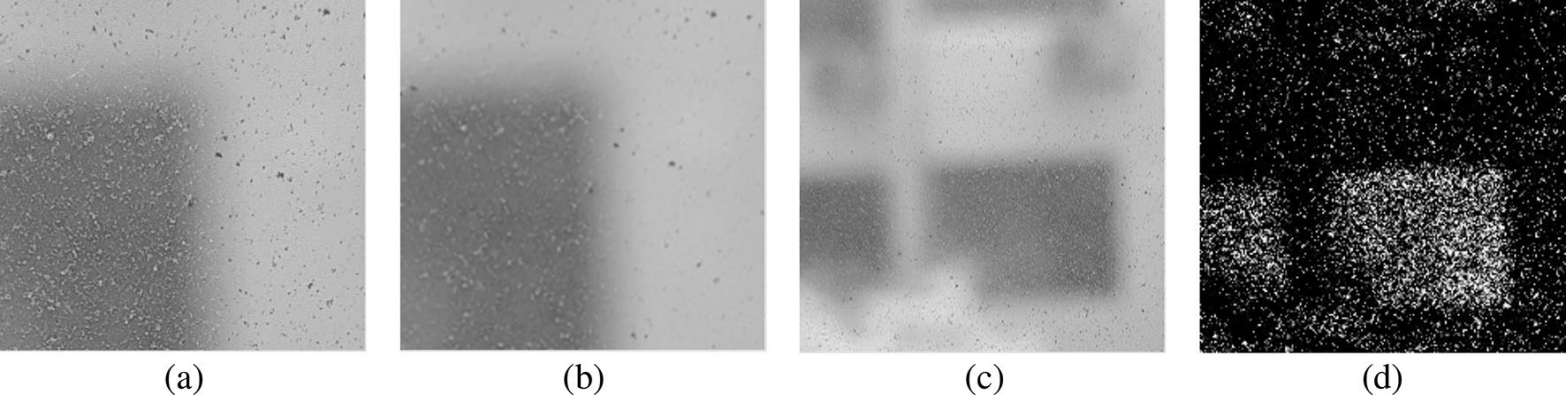
Example image of dust on a window. As the focus is close to the window, the dust is sharp and the background is blurred. Because the color of the background is different, detection works well in the area where the background and dust contrast well. This makes the dust detection result in cluster form. (**a**) shows an enlarged original image with contrasting background color. (**b**) shows the median filtered image-a. (**c**) shows the original image. (**d**) shows the binary image generated.

## Overall two-stage structure

Our proposed method is divided into two stages: dust separation and true density estimation. The first stage is to separate sections that look like dust-as a binary image-from images of a translucent glass window. The second stage is to estimate the true dust density of the entire window by using the binary image obtained earlier.

### Dust separation

Dust particles are small. Most cameras set the focal point close to the window surface so as to capture these small dust particles. This would focus the camera on the translucent window surface and sharpen the dust particles. The authors concentrated on the fact that the background was blurry and the dust particles were sharp. Therefore, this problem was solved using edge detection or a noise filtering method, as shown in Fig. 1, the details of which are discussed in “Dust separation stage”.

### True density estimation

Most of the dust adhering to windows in tall buildings has a uniform density. This is based on the fact that the small particles are scattered. However, depending on the color of the background, there are areas where dust particles are more easily visible than others. This means that the camera cannot display all of the dust particles on the screen. Therefore, it is necessary to estimate the true dust density of all dust particles using just some of them. The authors approached this problem by proposing an area representing the true density through clustering, focusing on the fact that the areas where dust particles were clearly captured appear in the form of a cluster. The details are presented in “True density estimation stage”.

## Test environment

### Test bench

**Figure 2:**
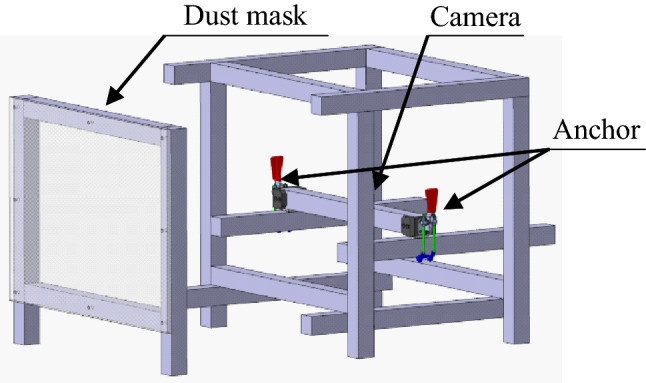
Test bench overview.

The test bench consisted of two parts. First, a fixture that held the camera was located in the middle of a cube made of aluminum profiles. The camera used was a Basler acA1440-220uc USB 3.0 color camera. A reference specimen that simulated dust particles was held in front of the camera. This test bench was placed in various environments, and the experiment was conducted using the acquired images. Figure 2 shows the structure of the test bench.

**Figure 3:**
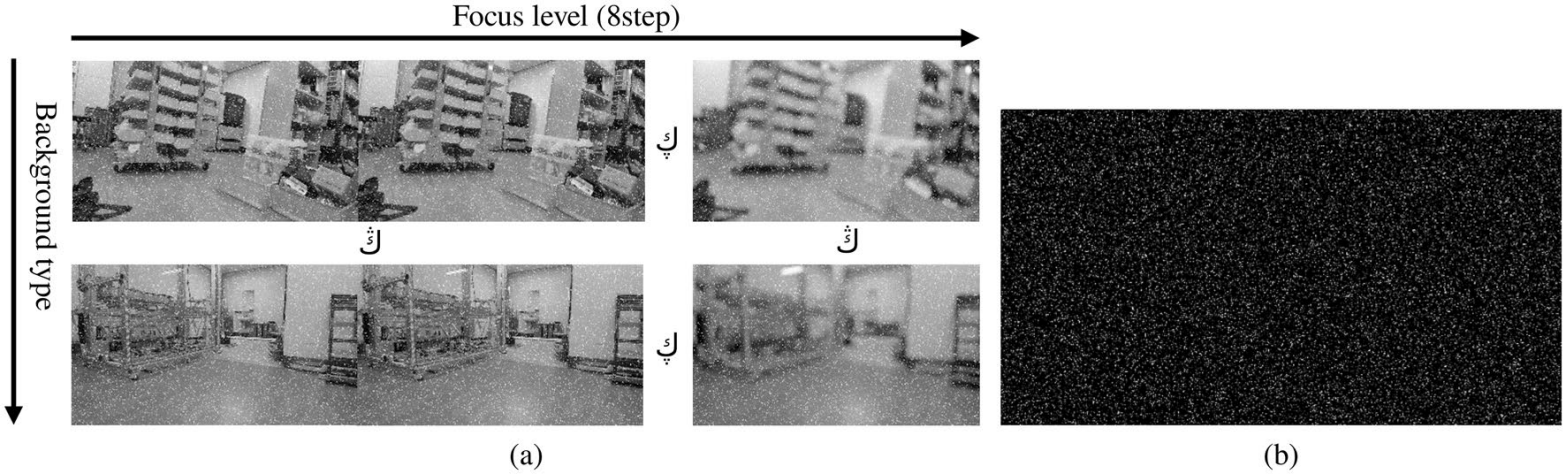
Image set and dust mask. (**a**) image set with two axes: focus level & background type. (**b**) the example of generated artificial dust mask.

### Reference specimen

If the experiment was conducted using a window with real dust particles, it would not be possible to determine the true dust density. Consequently, we would be unable to analyze the experimental results. Therefore, a substitute with a similar appearance to that of a window with real dust had to be developed. In this study, we used a transparent acrylic plate with laser-engraved dots on it. This was done to control the true dust density of the specimen. To simulate the dust particles, a random scattered pattern was used, simulating the shape of a scatter pattern with a uniform density of actual dust particles.

## Dust separation stage

When the camera is focused on the dust particles, the background is out of focus, as shown in Fig. 1a. At this point, the dust particles to be separated are sharp, and the background, which is to be ignored, is blurry. To distinguish between the two, an edge detection method or a noise filter can be used. We experimented using various methods. For edge detection, the Sobel^12^ and Prewitt^13^ methods were evaluated. Gaussian blur^16^ and median filter^14^ techniques were used as noise filtering methods. The edge detection method detects sharp dust pixels by considering them to be a type of boundary line. The noise filtering method considers dust pixels to be noise and eliminates them, enabling dust pixels to be detected using image differencing techniques.

Different methods create different detection images. The edge detection method conducts thresholding on the image on which the method was applied. The noise filtering method conducts thresholding on the difference between the original image and the noise-filtered image. To determine which method to use, we defined two scores. The dust separation score (*DSS*) is a score for how well the method separates dust particles. The background ignore score (*BIS*) is a score for how well the method produces a good *DSS* with only a small degree of background blur. To calculate the score, we synthesized a randomly generated dust mask using various blurry backgrounds, after which, four methods were compared using the corresponding images.

### Dust mask & blurry background image set

For the test, we needed a randomly generated dust mask to overlay the 720p image. For an image with a resolution of 1280 720 pixels, we generated dust particles with random size, blur, and grayscale at uniform random points number of 2304, 9216, 20736, 36864. The size range was [0   1], the blur sigma (Gaussian filter size) range was [0   3] and [0  5], and the grayscale range was [50   255]. An image set was created by combining this dust mask with background images of various background types and focus levels. MATLAB was used for generating and converging test image sets.

The image set to be used for the test was created by synthesizing an image taken by setting only the focus differently at the same position as the dust mask made in this way. Once the true dust pixel information and the sharpness information of the background image were known, the *DSS* and *BIS* could be calculated. Figure 3 shows the structure of the image set.

**Figure 4:**
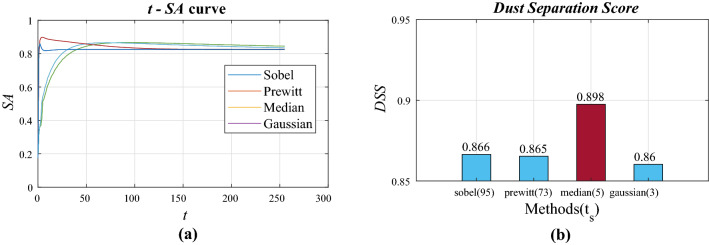
*t*-*SA* curve shows each method’s *SA* for the threshold range of [0   255]. Median filter obtained the highest *DSS*.

### Dust separation score

First, it must be determined how well the method distinguished between the dust particles and the background. The definition of the separation accuracy (*SA*) of one image, comparing the image detected through our method against the dust mask, with image size of $n \times m$ is as follows:

1 $$\begin{aligned} SA(I,M,T))=\frac{\sum _{n}^{i=1}\sum _{m}^{j=1}T(I_{i,j},t) \bigoplus T(M_{i,j},1)}{n\times m} \end{aligned}$$

*t*: : target threshold, [0 255] range integer.
$$I_{i,j}$$
: detection image using our methods. Pixel value of position (i,j).
$$M_{i,j}$$
: dust mask image, pixel value of position (i,j).
*T*(*p*, *t*): : Thresholding function 1 (true) when $p\ge t$, else is 0 (false).
$$\bigoplus $$
: XOR operation in boolean value

SA has a range of [0   1], a value closer to 1 indicates that the dust pixel has been successfully separated. After calculating this index for all images in the image set, the highest average values of *t* and *SA* at that time are called $t_s$ (separation threshold) and *DSS*, respectively.

Therefore, the definition of DSS is as follows:

2 $$\begin{aligned} DSS= & {} mean(\sum SA(I,M,t_S)) \end{aligned}$$

3 $$\begin{aligned} t_s= & {} max(mean(\sum SA(I,M,t)) \end{aligned}$$

Figure 4 shows the $t-SA$ plot and *DSS* for the four methods. The median filter (filter size 5) showed the best *DSS* with a value of 0.898. Next, the Sobel and Prewitt filters had a similar *DSS* of 0.866 and 0.865. Finally the Gaussian filter showed the lowest *DSS*, 0.86.

As the threshold value increases, more detected pixels are filtered, so that the image converges to an image with all pixel values of 0. Since the accuracy is evaluated by the XOR method, even if the part where the original value should be 0 is 0, it is scored. Therefore, it converges to the score for the empty part of the dust mask. It can be confirmed that for each of the four types of methods, as t approaches 255, it converges toward a certain value. Sobel and prewitt filters showed lower values than the minimum *DSS* received even with a black screen. This means that no matter how well the threshold was set, it was not possible to reduce the misconception that there is dust where there is no dust. However, the gaussian filter and the median filter showed higher *DSS* than the minimum *DSS* at a specific threshold value, which means that the noise reduction method has a better overall effect than the boundary detection method. Among them, the median filter showed the best *DSS*.

**Figure 5:**
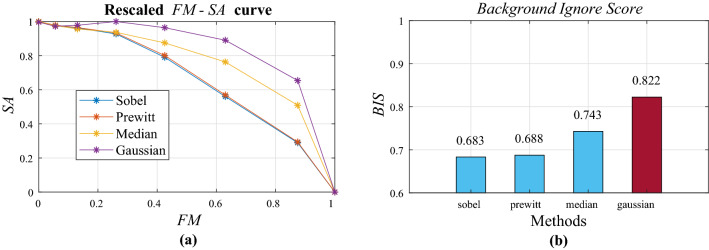
Rescaled *FM*-*SA* curve shows *SA* dropping as *FM* increases. The Gaussian method obtained the highest *BIS*.

### Background ignore score

For each edge detection algorithm, a test was conducted to determine how blurry the background needed to be before it could not be detected. This depends on the camera, but the background must focused on to use edge detection. At this stage, the larger the minimum blur level that ignores the background, the larger the minimum distance between the transparent window and the background objects. When detecting the background, the result is different as a part other than the desired dust particles is detected. Therefore, the best method to use is a method that can separate the background and dust pixels with the least blur. In this study, the sharpness of the background was measured using a frequency modulation measuring image quality (FM) method^18^.

We set the sharpness of the background measured through FM on the x-axis. Using the previously introduced $t_s$, an FM-SA plot was created by placing $SA(I,M,t_s)$ as the y-axis. At this stage, the image used is the same background image with different focus levels. Subsequently, the *x* and *y* axes are rescaled to a range of [0   1]. For rescaling, the rescale function of MATLAB was used. Consequently, the definition of the *BIS* is as follows:

4 $$\begin{aligned} BIS=\sum _{b_{type}}^{b=1}\int _{f=f_{step}}^{f=1}rescaledSA(I_{b,f},M,T_s)d FM(BI_{b,f})) \end{aligned}$$

*FM*(*I*): : sharpness measured value of image *I* by *FM*.
$$b_{type}$$
: number of background types in image set.
$$f_{step}$$
: number of focus level steps in image set.
$$I_{b,f}$$
: image at image set with background type *b*, focus level *f*.
$$BI_{b,f}$$
: image at background image set with background type *b*, focus level *f*.

Figure 5 shows the FM-SA plot and the BIS for each method. The Gaussian filter was the highest at 0.822, followed by the median filter at 0.741, and the Sobel and Prewitt filters were lower.

### Procedure of dust separation stage

Through a series of comparisons, *DSS* was in the order of Median> Gaussian> Sobel $\simeq$ Prewitt, and *BIS* was Gaussian> Median> Sobel $\simeq$ Prewitt. The BIS can be overcome by adjusting the camera’s focus and shooting environment, but the DSS is difficult to change. Consequently, a median filter was used.

The image processing procedure of the first stage using a median filter is as follows. The filter receives the original image as an input and outputs a binary image separated by dust.

1. Import original image *A*.
2. Create $A_gray$, i.e., a grayscale image of *A*.
3. Create $A_m$, i.e., a median filtered image of $A_gray$ with filter size 5.
4. Create $$A_{diff}$$ as $$abs(A_{gray} A_m)$$.
5. Create $$A_{dust}$$ as $$T(A_diff,t_{s,median}=6)$$.

In Fig. 1a,c represent $A_gray$, (b) denotes $A_m$, and (d) represents $A_dust$. The resulting binary image is used in the next stage, i.e., the true density estimation stage.

**Figure 6:**
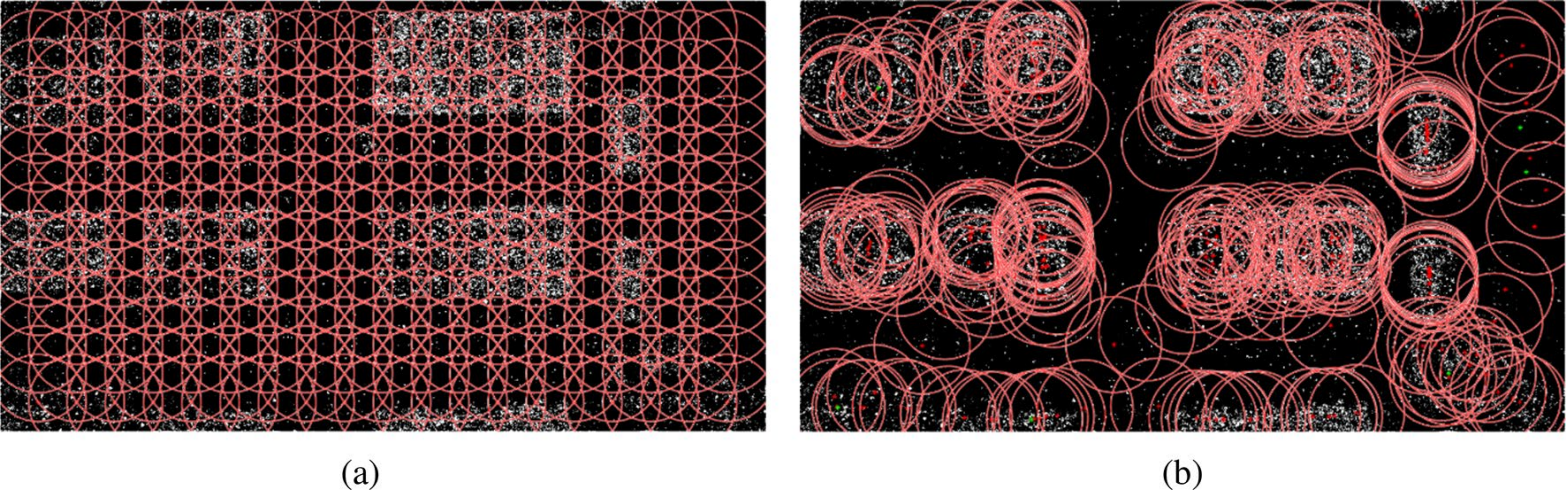
When mean-shift clustering is in progress, the red circle represents the window of each centroid, the green point represents the dead centroid, and the red point represents the alive centroid. (**a**) shows the initial position of the centroid. (**b**) is the state in the middle of clustering. It can be seen that the density is moving toward higher spaces.

## True density estimation stage

As can be seen in Fig. 1, the result of dust detection is largely aggregated into clusters depending on the background. Therefore, a proposal for an area of maximum density based on the dust binary image from the first stage is required. The detection area proposal proceeds through clustering, and the dust density of the proposed area is returned as the estimated dust density value. There are several types of clustering algorithms, such as the k-means^19,20^, DBScan^21^, and mean shift^17,22^ methods. We chose a mean shift approach using a flat kernel. This is because there is no need to predetermine the number of clusters. Moreover, if the initial positions of the centroids are in the form of a grid on the image, this approach can search for the global maximum density.

### Mean shift clustering

Clustering using mean shift has been reported previously^17^. Mean shift is an iterative algorithm that moves each centroid point to the average of the data adjacent to the corresponding area. In this study, a mean shift with a flat kernel was used for 2D Boolean data. At this stage, let the data set *S* to be the circle area radius of $\lambda$ and center is m. Then, the mean shift is expressed as follows:

5 $$\begin{aligned} F(x)=\left\{ \begin{matrix} 1 &{} if &{} \left\| x \right\| \le \lambda \\ 0 &{} if &{} \left\| x \right\| > \lambda \end{matrix}\right. \end{aligned}$$

6 $$\begin{aligned} m(x) = \frac{\sum _{s \in S}^{} F(s-x) \times s}{n(S)} \end{aligned}$$

7 $$\begin{aligned} D_m = \left\| m(x) - m \right\| \end{aligned}$$

*s*: : each pixel value of *S*
*F*(*x*): : flat kernel
$$\lambda $$
: window radius
$$D_m$$
: mean shift distance

This process is repeated for all centroids until convergence-that is, the centroids climb toward a higher density in the density space and stop at the local maximum with the largest density.

**Figure 7:**
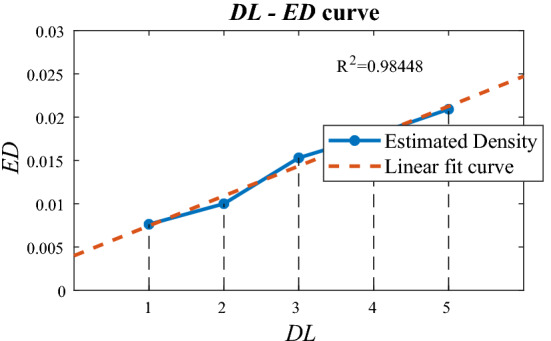
*DL*-*ED* curve of the dust density estimation stage. The *ED* and *DL* are directly proportional with a good coefficient of determination.

**Figure 8:**
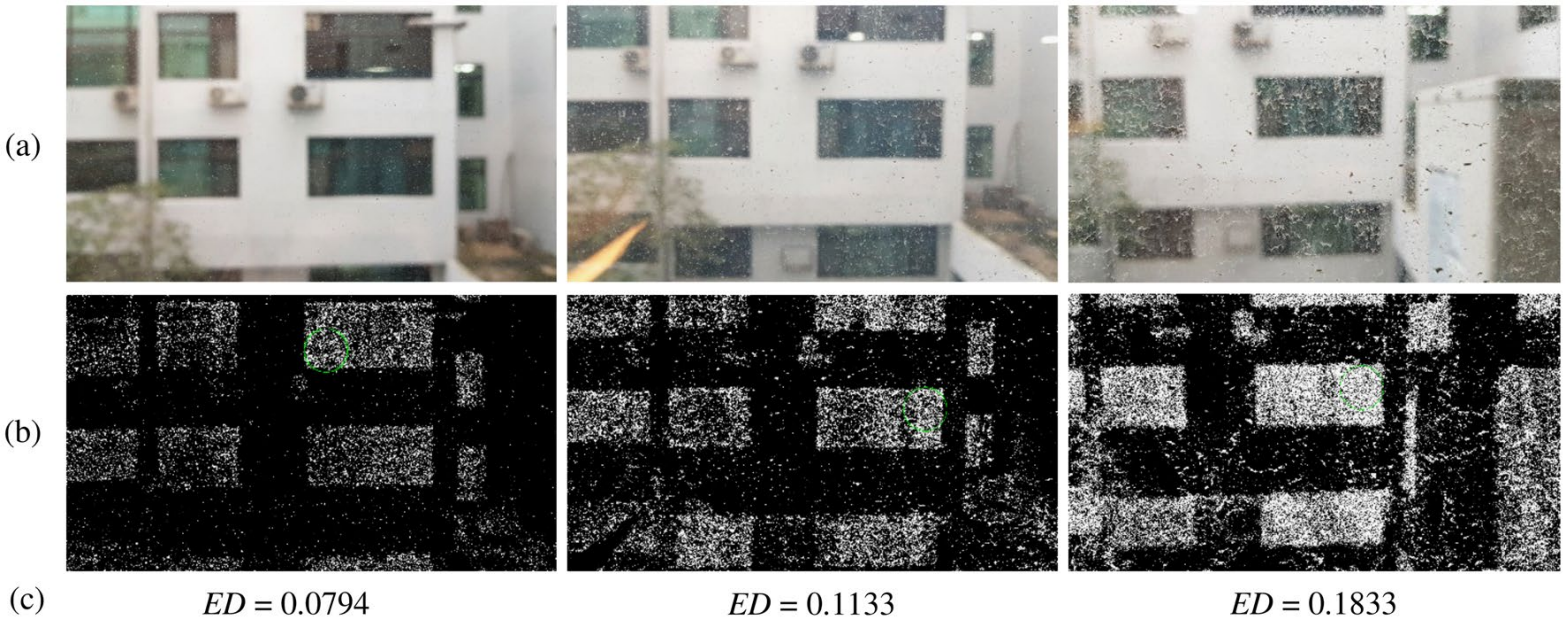
Result of applying the method of this study to real images. The more dust on the window, the higher the estimated dust density value. (**a**) is original image. (**b**) is separated dust and proposed region for dust density estimation. (**c**) is estimated density.

### Detection area proposal by mean-shift

In this study, mean shift clustering was applied to the density estimation stage by focusing on the property that mean shift clustering converges to a high density. We need the global maximum density, but one centroid will converge to the local maximum. To solve this problem, we uniformly arrange the initial positions of the centroids throughout the image to secure centroids that converge to the global maximum density. After setting the initial position in the form of a grid on the image, mean shift clustering is performed. When clustering is completed, the maximum density is estimated by sorting the density around each centroid in descending order.

The parameters used in this process are as follows.

1. *l*: grid length. The initial position of the centroid is arranged in the form of a grid, which is the length of one side of the grid. This was arbitrarily set to 40.
2. $\lambda$: window radius. The radius of the window is observed at one centroid. We set the diagonal length of the grid so that there is no empty space at the initial position. $\lambda = \sqrt{2}l$
3. $D_d$: dead distance. When the centroid has almost stopped moving, the point is assumed to be a dead point, further calculations are stopped, and the point is assumed to be the convergence point. Centroids moving below $D_d$ converted to the dead state.

At this time, the process of the density estimation stage using mean shift is as follows (moreover, this can be checked in Fig. 6).

1. Import $A_dust$ from the first stage.
2. Initialize centroids *C* in the form of a grid with parameter *l*.
3. Perform mean shift process with $A_dust$ using $\lambda , l, D_d$. Obtain converged *C*.
4. Obtain the top 3% of the density of each centroids in *C*.
5. Mean of 4) is the estimated dust density *d*.

There are two ways to perform the mean shift process. The first is a method of performing the mean shift of all centroids that are alive for each iteration. This makes all the points converge as a whole, but the calculation of centroids with a very small $D_m$ tends to slow the calculation of centroids with a large $D_m$. If calculations cannot be conducted until the end and are stopped prematurely, the convergence progress of each centroid will be different. The second way to perform this method is to use a priority queue. Centroids are placed in the priority queue, and only the centroid with the largest $D_m$ is dequeued and processed. In this process, if $D_m$ is smaller than $D_d$, it is processed as the corresponding dead centroid. In other cases, it is the alive centroid, and thus the $D_m$ updated is enqueued in the priority queue once again. This process continues until the priority queue is empty. With this method, even if the calculation process is stopped in the middle, the convergence progress of each centroid remains similar. The pseudo code for each case can be found in Appendix 1. In this study, the first method that calculating the total mean shift for each iteration was used.

### Estimation accuracy test

To test the estimation accuracy of the second stage, the test bench of Fig. 2 was used. We chose transparent acrylic as the dust mask specimen. The dots were engraved using a laser cutter, and this acrylic dust specimen was used for testing in an actual shooting environment. When the dot density of the acrylic dust specimen with the lowest density was 1, it had a density level (*DL*) of 1, 2, 3, 4, and 5. Appendix 2 shows each *DL* demonstration engraved on an acrylic plate. Next, using a test bench, 12 photos were taken with random backgrounds for one dust mask.

After all dust density estimations were performed for one *DL*, the results were averaged; this average was defined as the estimated density (*ED*) of the *DL*. Consequently, the result was validated, as shown in Fig. 7. As the *DL* of the acrylic dust specimen increased, the *ED* also increased at a similar rate. A linear fit was performed using the least squares method, and the coefficient of determination was 0.984. In addition, by processing with a picture of an actual window, it was also confirmed that the amount of dust and the dust estimation result were proportional, as shown in Fig. 8.

Weather conditions were not considered. This is because the exterior wall cleaning robot is not operated in snowy and rainy weather for safety reasons. Especially in rainy weather, there is no need to proceed with cleaning. Sunny and cloudy weather conditions are the difference in whether strong direct sunlight is reflected on the glass window, and if a cover that blocks direct sunlight is used on the robot’s camera part, the difference becomes meaningless. Therefore, this experiment was conducted focusing on the brightness condition rather than the weather.

## Conclusion & discussion

In this study, a method for estimating the dust density of a translucent glass window was developed using simple image processing methods. Different from previous study^8^, this method has more free brightness condition for implementation in real-world facade cleaning robot. A dust separation stage was performed through a median filter, and dust density estimation was performed through mean shift clustering. Experiments conducted with a test bench showed that the estimated dust density was directly proportional to the true dust density. Moreover, a good coefficient of determinant of 0.984 was obtained as a result of the linear fit. This method is relatively free of global brightness and background influence and can operate under various brightness conditions, such as on the exterior wall of a building. This method can be applied if the camera is focused on the dust on the glass surface, and the background is sufficiently blurred. Moreover, a low-quality camera may be used, as long as a sufficient shutter speed can be obtained so that dust does not itself become blurred when shooting on the move. However, this method does have some limitations. First, if there is an object too close to the window, the object is clearly visible even if the camera focuses on the window surface. In this case, edge detection of the object occurs and greatly disturbs the dust estimation result. Second, if a light source is captured directly by the camera or reflected off the window, the background is captured with very sharp edges, regardless of the distance from the camera. This also has a significant influence on the dust estimation results.

## Data Availability

All of the codes used in this paper can be found at github public repository: https://github.com/jisuk500/Translucent-Dust-Detection.
